# P-454. An Analysis of an Evolving Malignancy Profile in an Urban, Predominantly African-American Population Living with HIV

**DOI:** 10.1093/ofid/ofae631.654

**Published:** 2025-01-29

**Authors:** Melissa K Rallo, Nicholas Leahy, David J Riedel

**Affiliations:** Institute of Human Virology at the University of Maryland School of Medicine, Baltimore, Maryland; University of Maryland Institute of Human Virology, Baltimore, Maryland; Institute of Human Virology, University of Maryland School of Medicine, Baltimore, MD

## Abstract

**Background:**

It is well known that HIV increases the risk of certain malignancies, including AIDS-defining cancers (ADCs) such as Kaposi Sarcoma, non-Hodgkin Lymphoma, and invasive cervical cancer. While the incidence of effective antiretroviral treatment (ART) has allowed the incidence of ADCs to decline, HIV-infected individuals are still at a higher risk for non-AIDS-defining cancers (NADC). A 2014 study conducted at the University of Maryland Medical Center (UMMC) showed that while HIV-infected patients presented with late-stage cancer and significant mortality, ART had a significant mortality benefit. While this study was pivotal, it’s important to consider how this malignancy profile continues to change. The purpose of this study is to characterize the numbers and types of cancers, patient demographics, ARTs, and mortality among HIV-infected patients in an urban population in order to contrast with data from an older cohort of data.

**Methods:**

A retrospective cohort study was used to review 301 HIV-infected patients diagnosed with invasive cancer from January 1st, 2015 to December 31st, 2022 who were managed at UMMC.

**Results:**

There were 322 cases of invasive cancer in this population. Patients were predominantly African American (81%) and male (61%). The majority of cases involved patients who were compliant with an ART regimen when they presented initially for diagnosis (79%) with a subset of patients meeting the criteria for AIDS (CD4 < 200) at the time of cancer diagnosis (25%). The overall incidence of stage 3 or 4 cancer in the population was 45%, and 58% among HIV-infected patients who met the clinical criteria for AIDS. There were 139 deaths with a majority of the causes of death attributed to cancer progression or infection (68%).

**Conclusion:**

An evolving ART regimen profile is continuing to mold the malignancy composition of patients living with HIV. When the current retrospective cohort was contrasted with the older cohort from 2000 to 2010, there were a few key changes – most notably a higher composition of females, a higher percentage of those with a documented ART regimen at the time of diagnosis, and a lower proportion of malignancies at diagnosis meeting Stage 3 or 4 criteria.

**Disclosures:**

**All Authors**: No reported disclosures

